# Antitumor Effects of Baicalein and Its Mechanism via TGF*β* Pathway in Cervical Cancer HeLa Cells

**DOI:** 10.1155/2021/5527190

**Published:** 2021-03-11

**Authors:** Gang Yu, Lizhen Chen, Yuanhua Hu, Zhen Yuan, Yao Luo, Yuanhuan Xiong

**Affiliations:** ^1^Jiangxi Provincial People's Hospital, Nanchang, China; ^2^Jingdezhen No. 1 People's Hospital, Jingdezhen, China; ^3^Shunde Maternal and Child Health Hospital, Guangdong Medical University, Foshan, China; ^4^Yichun Institute of Vocational Technology, Yichun, China; ^5^Medical College of Nanchang University, Nanchang, China; ^6^Jiangxi the Fifth People's Hospital, Nanchang, China

## Abstract

**Background:**

Due to dual-regulating carcinogenesis, the TGF*β* pathway is an ideal and alternative tumor target. Natural flavonoids possess the similar structures to estrogen and could exert an important benefit to cervical cancer. The present study aimed to screen the inhibitor of TGF*β* pathway from natural flavonoids and evaluate the function and mechanism of the TGF*β* pathway inhibitor on cervical cancer.

**Materials and Methods:**

The cervical cancer HeLa cells were firstly treated with different flavonoids and probed by western blot for screening the inhibitor of TGF*β* pathway. And then, the effect of the identified inhibitor on cell proliferation was studied by CCK-8 and clone formation assay. Then, RT-PCR and western blot assay were performed to evaluate the effect of identified inhibitor on mTOR/p70S6K pathway, and the cell migration and EMT pathway were also examined using scratching analysis and western blot assay. Finally, the role of TGF*β* was assessed via the classic inhibitor of TGF*β*/SMAD pathway.

**Results:**

Screening data by western blot assay showed that baicalein displayed the best inhibitor effect on TGF*β* expression. CCK-8 and clone formation assay showed that baicalein inhibited the cell proliferation and clone cell number. RT-PCR and western bolt for probing mTOR, p70S6K, and 4EBP1 revealed that baicalein could suppress their expression and phosphorylation. The scratching analysis and western blot assay displayed that baicalein inhibited the cell migration and EMT progression in HeLa. The use of SB431542, a TGF*β* inhibitor, revealed that TGF*β* was crucial to baicalein-regulating cell proliferation and migration in HeLa cells.

**Conclusion:**

Baicalein, a medicine agent screened from natural flavonoids targeting TGF*β* pathway, could suppress mTOR/p70S6K pathway-mediated cell proliferation and EMT pathway-related migration via TGF*β* pathway in cervical cancer HeLa cells.

## 1. Introduction

Cervical cancer ranked the third most frequent carcinoma in women all over the world, and there are more than 500,000 increasing incidents year by year. It accounts for approximately 275 100 cancer-related deaths annually [1–3]. Although the incidences and deaths of cervical cancer in developed countries are decreasing greatly, it remains the leading cause of cancer-associated deaths in female from the undeveloped region [4, 5]. In China, the morbidity and mortality of cervical cancer is evidently increasing, and it has contributed to the third leading cause of cancer deaths [6]. Currently, many antitumor agents have been reported and applied to the treatment of cervical cancer [7, 8]. However, the effective medicine for cervical cancer remains a challenge in the clinic. Hence, it is an urgent need to identify novel and potential candidates for the treatment of cervical cancer.

Natural products have been used in the folk medicine around the world for thousands of years. Due to the historically validated benefits to health and the rich chemical ingredients, the natural products have been becoming the material sources of modern medicines [9]. According to statistics, there are approximately 65% of modern pharmaceutical agents approved by the American Food and Drug Administration (FDA) deriving from the ingredients of natural products [10, 11]. Among these complex ingredients of natural products, flavonoid is one kind of molecule with the similar structure to estrogen playing an indispensable role in the female physiology [12, 13]. Emerging evidences show that flavonoids could exert many benefit effects on cervical cancer. Liu et al. reported that some dietary flavonoids could inhibit invasion of cervical cancer via epithelial-mesenchymal transition signaling [14]. In another study, icariin displayed the inhibition effect on the growth of human cervical cancer cells by targeting the mTOR/PI3K/AKT signaling pathway [15]. This information reveals that flavonoid could be the important source of potential agents to treat cervical cancer.

The transforming growth factor (TGF*β*) signal pathway is considered as a dual-regulator of carcinogenesis [16, 17]. At the early stages of tumorigenesis, the TGF*β* signal pathway serves as a tumor inhibitor through promoting the cell apoptosis of premalignant tissues. However, at the advanced stages of tumorigenesis, TGF*β* signal pathway began to lose the tumor inhibitory function with the oncogenic mutations of tumor cells and following turning into the tumor promotor via moving the tumor cells to subject epithelial-mesenchymal transition (EMT) and caused the tumor cells metastasis. The paradoxical role of TGF*β* signal pathway in the different stages of carcinogenesis provides an extremely potential chance for drug development [18, 19]. Several evidences have verified that there are many compounds, including SB-431242, SD208, LY2109761, and IN-1130, exerting the antitumor activity via the TGF*β* signal pathway [18]. Therefore, the TGF*β* signal pathway is an ideal and alternative target for the development of antitumor medicine.

Here, we aimed to identify the potential candidate molecules targeting the TGF*β* signal pathway for the treatment to cervical cancer from the natural flavonoids and elucidate the mechanism of the candidate treating against cervical cancer. The present study would provide an alternative therapy to cervical cancer and enrich the application of flavonoids.

## 2. Materials and Methods

### 2.1. Cell Culture

The human cervical cancer HeLa cells were cultured in RPMI-1640 medium (Hyclone, USA) supplemented with 10% fetal bovine serum (FBS, Excell Bio, China) in a humidified atmosphere of 5% CO_2_ at 37°C.

### 2.2. Western Blot

When the HeLa cells reached the 80–90% confluence, they were seeded and cultured for 12 hours. At the 12^th^ hour, 20 *μM* or 50 *μM* flavonoids (Chengdu Biopurify, China) were added into the well of cell cultured plates and cultured for another 12 hours. At the 24^th^ hour, the cells were lysed with NP40 lysis buffer (Solarbio, China), and the total protein was prepared and boiled for degeneration. 30 *μ*g total protein was loaded into the SDS-PAGE and run for 2 hours. After 2 hours, the protein samples in gels were transferred to the PVDF membrane (Millipore, USA), blocked with 5% skim milk, and then were incubated with the primary antibody of TGF*β*, mTOR, p70S6K, 4EBP1, p-mTOR, p-p70S6K, p-4EBP1, E-cadherin, Snail, FAK, and p-FAK (Proteinteck, China) for immunoreaction, then washed, incubated with secondary antibody, and visualized using an enhanced chemiluminescence system (Proteinteck, China).

### 2.3. CCK-8 Assay

When the HeLa cells reached the 80–90% confluence, they were seeded and cultured for 12 hours. At the 12^th^ hour, the cells were added with 20 *μM* or 50 *μM* baicalein and cultured for six time of 12, 24, 36, 48, 60, and 72 hours. At the indicated time, CCK-8 solutions (Beyotime, China) were added into the well of cell cultured plates and cultured for another 1 hour. The absorbance of cells at 450 nm was detected using microplate reader (Bio-Tek, USA). The proliferation ratio was calculated as

(1) $$\begin{array}{c} \text{proliferation ratio}\left(\%\right)=\left(\frac{{\text{OD}}_{\text{sample}}-{\text{OD}}_{\text{blank}}}{{\text{OD}}_{\text{control}}-{\text{OD}}_{\text{blank}}}\right)\times 100\%. \end{array}$$

### 2.4. Clone Formation

When the HeLa cells reached the 70–80% confluence, they were seeded and cultured for 12 hours. At the 12^th^ hour, the cells were added with 20 *μM* or 50 *μM* baicalein and cultured for one week. After one week, the cells were washed with PBS for twice, fixed with 4% paraformaldehyde for 5 minutes, stained with Gimsa (Shinoda, China) for 20 minutes, and photos were taken using camera.

### 2.5. RT-PCR

When the HeLa cells reached the 80–90% confluence, they were seeded and cultured for 12 hours. At the 12^th^ hour, the cells were added with 20 *μM* or 50 *μM* baicalein and cultured for another 12 hours. At the 24^th^ hour, the cells were lysed with trizol (Thermo, USA), and total RNA was extracted. The extracted RNA was reversely transcribed into the first-strand cDNA (Tiangen, China), and then PCR amplification of target genes of mTOR, p70S6K, and 4EBP1 was carried out in the PCR amplifier (Bio-Rad, USA). The amplification products were detected via gel electrophoresis. The primer sequences of mTOR, p70S6K, and 4EBP1 are as follows [20]:

  mTOR: F: GCCGCAUUGUCUCUAUCAATT; R: UUGAUAGAGACAAUGCGGCTT
  p70S6K: F: ACTTCTGGCTCGAAAGGTGG; R: TTGAGTCATCTGGGCTGTCG
  4EBP1: F: CCTTTCCGGGACTTTCGCTTT; R: GCAGAATCCAGGTGGCAACA

### 2.6. Scratch Test

When the HeLa cells reached the 80–90% confluence, they were seeded into the 6-wells cell cultured plates which were premarked in the bottom of plated with the line as a reference for image acquisition. After 12 hours, the seeded cells were scratched with 10 *μL* pipette tip to form a cell-free area and treated with 20 *μM* or 50 *μM* baicalein for another 24 hours. At the coming of the schedule time, the cells were imaged using microscope (Olympus, Japan).

### 2.7. Statistics Analysis

The statistical analysis was carried out using SPSS software (IBM, USA). The measurement data were presented as the mean ± SD. Comparisons between two groups were performed by the Student's *t*-test. The level of *p* < 0.05 was considered to be statically differences.

## 3. Results

### 3.1. Identification of TGF*β* Inhibitor

**Figure 1:**
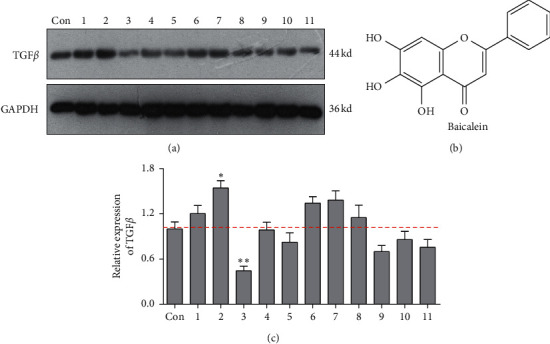
Baicalein is an inhibitor of TGF*β* pathway. (a) Western blot assay for probing TGF*β* expression. (b) The quantitative analysis of TGF*β* expression. (c) The structure of baicalein. ^*∗*^*p* < 0.05 and ^*∗∗*^*p* < 0.01 vs. control group.

To screen the inhibitor of TGF*β* pathway, the expression of TGF*β* in human cervical carcinoma HeLa cells exposed to the series of natural flavonoids were probed by western blotting assay. As displayed in Figure 1, there were five flavonoids weakly inducing the TGF*β* expression and three flavonoids inhibiting the TGF*β* expression (Figure 1(a)). Among the inhibitors, flavonoids 3 of baicalein exhibited the most evident inhibitory effect on TGF*β* expression level (Figure 1(b), ^*∗∗*^*p* < 0.01 vs. control group) and SMAD2/3 phosphorylation (Supplementary Figure S1). Its structure is shown in Figure 1(c). The result demonstrated that baicalein might be a potential inhibitor of TGF*β* pathway and would become the object for the following study in the present research.

### 3.2. Effect of Baicalein on Cell Proliferation in HeLa Cells

**Figure 2:**
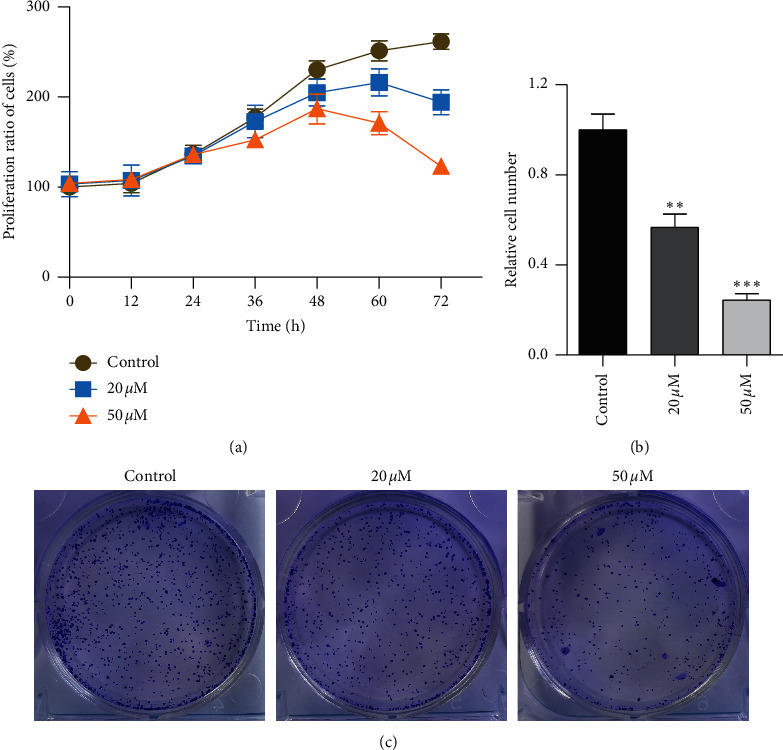
Baicalein suppresses the cell proliferation in HeLa cells. (a) CCK-8 assay for the curve of cell proliferation. (b) Colon formation assay for the cell proliferation. (c) The quantitative analysis of relative clone cell number. ^*∗∗*^*p* < 0.01 and ^*∗∗∗*^*p* < 0.001 vs. control group.

The unlimited proliferation is the symbol events of tumor growth. To confirm the effect of TGF*β* pathway inhibitor of baicalein on cervical cancer, CCK-8 and clone formation assay were used to examine the cell proliferation in human cervical cancer HeLa cells. The proliferation curve in Figure 1(a) showed that the proliferation ratio of cells exposed to baicalein displayed a time-dependent decrease from 0 to 72 hours. At the 72^nd^ hour, the proliferation ratio of 20 *μM* and 50 *μM* baicalein was, respectively, 72.1% ± 5.1% and 45.7% ± 2.5%, demonstrating a concentration-dependent effect. Furthermore, the images of clone formation showed that the relative cell number of baicalein at 20 and 50 *μM* was significantly inhibited (Figure 2(b) and 2(c), ^*∗∗*^*p* < 0.01 for 20 *μM* baicalein and ^*∗∗∗*^*p* < 0.001 for 50 *μM* vs. control group). The similar data were observed in SKG IIIa cells (Supplementary Figure S2). These results demonstrated that baicalein could suppress the cell proliferation in HeLa cells.

### 3.3. Effect of Baicalein on mRNA Expression of mTOR, p70S6K, and 4EBP1 in HeLa Cells

**Figure 3:**
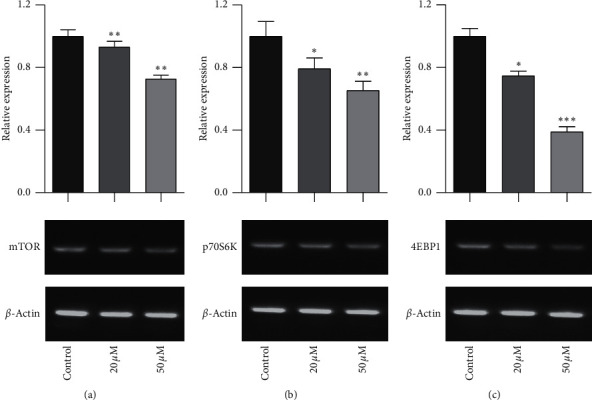
Baicalein inhibits the mRNA expression of mTOR (a), p70S6K, (b) and 4EBP1 (c). ^*∗*^*p* < 0.05, ^*∗∗*^*p* < 0.01, and ^*∗∗∗*^*p* < 0.001 vs. control group.

The mTOR/p70S6K signal pathway is well known to regulate cell proliferation. To evaluate the relationship of mTOR/p70S6K pathway with the antiproliferation effect of baicalein on cervical cancer, the mRNA level of mTOR, p70S6K, and 4EBP1 was analyzed with RT-PCR. As seen in Figure 3, baicalein inhibited the mRNA expression of mTOR (Figure 3(a)), p70S6K (Figure 3(b)), and 4EBP1 (Figure 3(c)) at different degrees in HeLa cells, and the high concentration of 50 *μM* baicalein displayed all statistical differences compared with that in control group (^*∗∗*^*p* < 0.01 for mTOR, ^*∗*^*p* < 0.05 for p70S6K, and ^*∗∗∗*^*p* < 0.001 for 4EBP1). The results indicated that baicalein might suppress the mTOR/p70S6K signal pathway in HeLa cells.

### 3.4. Effect of Baicalein on Protein Expression and Phosphorylation of mTOR, p70S6K, and 4EBP1 in HeLa Cells

**Figure 4:**
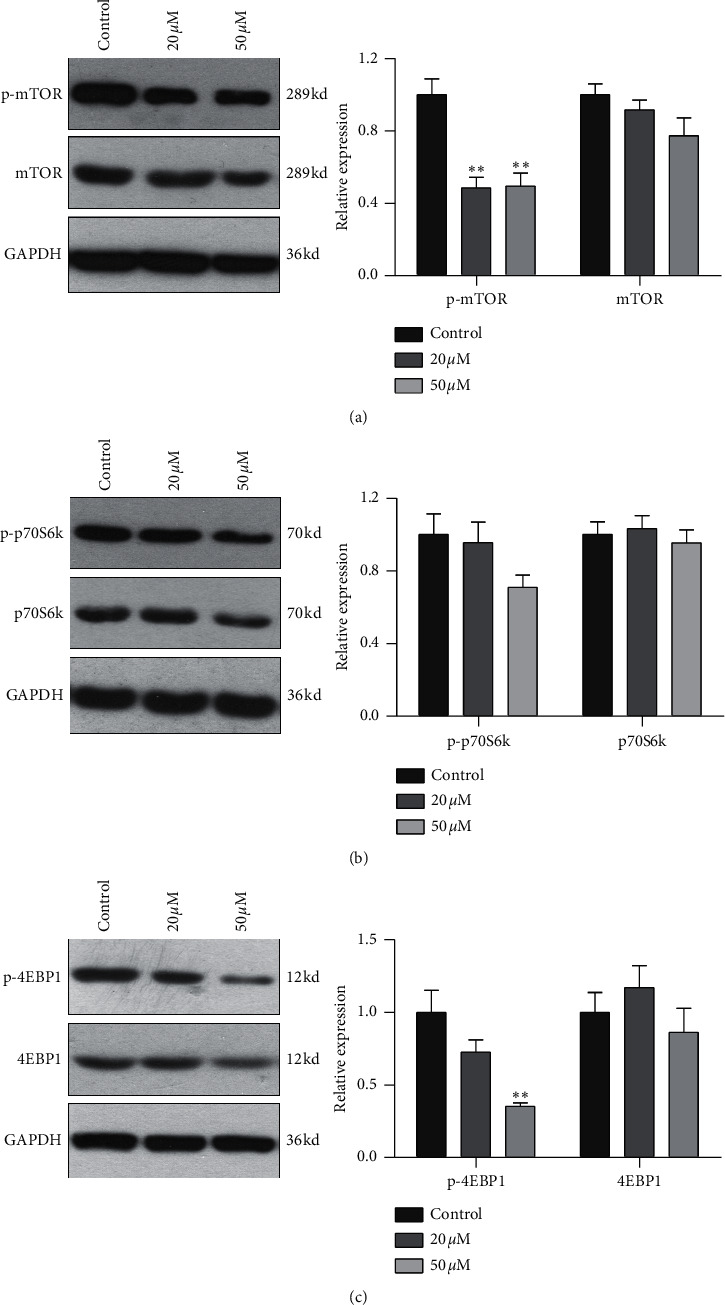
Baicalein inhibits the phosphorylation level of mTOR (a), p70S6K, (b) and 4EBP1 (c). ^*∗∗*^*p* < 0.01 vs. control group.

To further confirm the relationship of mTOR/p70S6K pathway with the antiproliferation effect of baicalein, western blot was employed to probe the protein expression and phosphorylation of mTOR, p70S6K and 4EBP1 in HeLa. As shown in Figure 4, both of 20 *μM* and 50 *μM* baicalein evidently inhibited the phosphorylation of mTOR (^*∗∗*^*p* < 0.01 for 20 *μM* and 50 *μM* baicalein vs. control group), 50 *μM* baicalein weakly inhibited the phosphorylation of p70S6K but no statistical differences, and 50 *μM* baicalein evidently inhibit the phosphorylation of 4EBP1. Meanwhile, 50 *μM* baicalein displayed a litter inhibitory effect on the expression of the three proteins. The similar effect by baicalein was observed in SKG IIIa cells (Supplementary Figure S3). Combined with the RT-PCR data, the results revealed that mTOR/p70S6K signal pathway may be in part contributed to the antiproliferation effect of baicalein in HeLa cells.

### 3.5. Effect of Baicalein on Cell Migration in HeLa Cells

**Figure 5:**
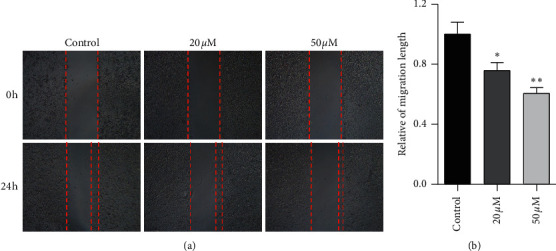
Baicalein suppresses the cell migration in HeLa cells. (a) Scratch assay for examining the cell migration. (b) The quantitative analysis for the relative migration length. ^*∗*^*p* < 0.05 and ^*∗∗*^*p* < 0.01 vs. control group.

Tumor cells migration is considered as one of the leading deaths causes of advanced carcinoma. To study the effect of baicalein on cervical cancer metastasis, scratch test was carried out to assess the cell migration ability. The images of HeLa cells displayed that, after the treatment with baicalein for 24 hours, the width of cells-free area is wider than that in the control group (Figure 5(a)). The statistical analysis showed that baicalein evidently inhibited the cell migration of HeLa cells (^*∗*^*p* < 0.05 for 20 *μM* and ^*∗∗*^*p* < 0.01 for 50 *μM* baicalein vs. control group. The results demonstrated that baicalein could suppress the HeLa cells migration.

### 3.6. Effect of Baicalein on EMT Pathway in HeLa Cells

**Figure 6:**
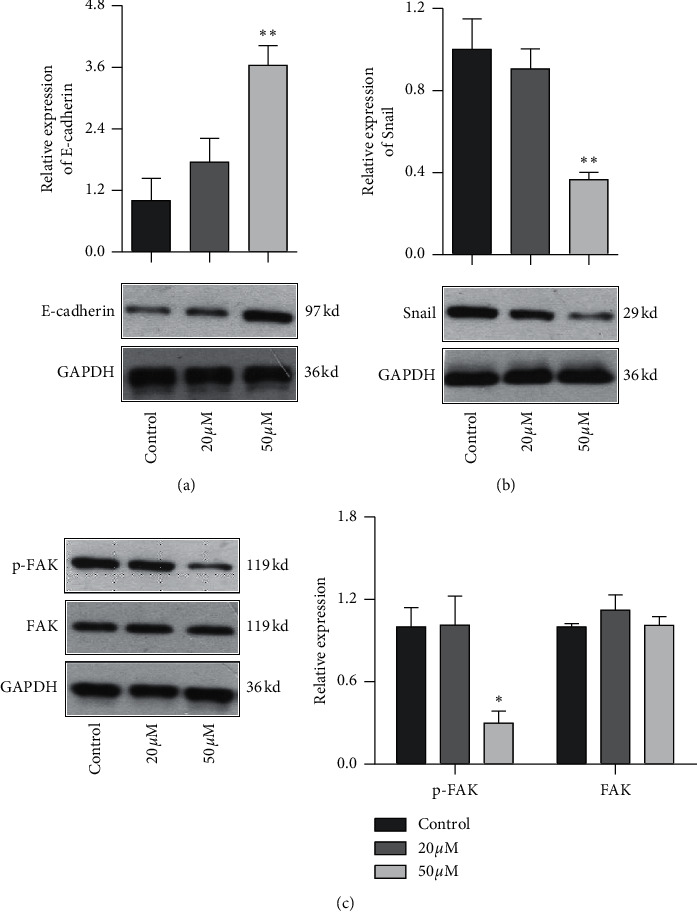
Baicalein suppresses the EMT pathway in HeLa cells. Western blot for probing the expression of E-cadherin (a), Snail (b), and FAK and p-FAK (c). ^*∗*^*p* < 0.05 and ^*∗∗*^*p* < 0.01 vs. control group.

EMT pathway is an essential step of tumor metastasis from primary location to target location. To clarify the effect of baicalein on EMT pathway, western blot was employed again to probe the expression of E-cadherin, Snail, and FAK and the phosphorylation of FAK. As seen in Figure 6, 50 *μM* baicalein induced the expression of E-cadherin (Figure 6(a), ^*∗∗*^*p* < 0.01 vs. control group) and inhibited the expression of Snail (Figure 6(b), ^*∗∗*^*p* < 0.01 vs. control group) and the phosphorylation of FAK (Figure 6(c), ^*∗*^*p* < 0.05 vs. control group); meanwhile, there were no changes in the three indicator of cells subjecting low concentration of 20 *μM* baicalein. These results demonstrated that baicalein could suppress the EMT pathway in cervical cancer HeLa cells.

### 3.7. The Role of TGF*β* in Baicalein Suppressing Cervical Cancer in HeLa Cells

**Figure 7:**
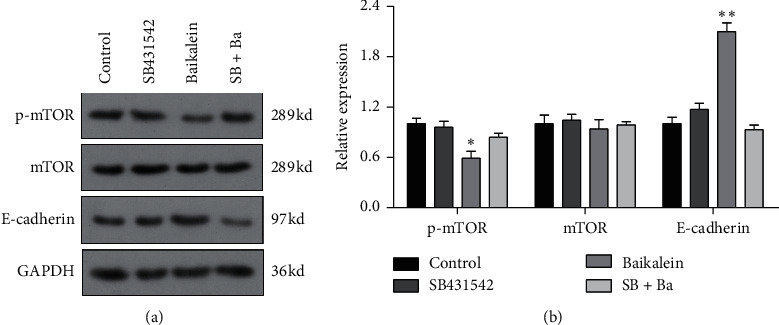
TGF*β* is crucial to baicalein-mediating proliferation and migration in HeLa cells. Western blot for probing the expression of p-mTOR, mTOR, and E-cadherin. ^*∗*^*p* < 0.05 and ^*∗∗*^*p* < 0.01 vs. control group.

In order to verify the role of TGF*β* in baicalein suppressing cervical cancer, SB431542, a TGF*β* inhibitor, was employed to further assess the effect of baicalein on the expression of p-mTOR, mTOR, and E-cadherin. As seen in Figure 7, the administration of only baicalein inhibited the phosphorylation of mTOR (^*∗*^*p* < 0.05 vs. control group) and induced the expression of E-cadherin (^*∗∗*^*p* < 0.01 vs. control group); however, the change trends were disappeared following the coadministration of baicalein and SB431542. These results demonstrated that TGF*β* could be crucial to baicalein-mediating cell proliferation and migration.

## 4. Discussion

Flavonoid is recognized as a kind of natural molecules widely existing in the plates, which has the similar chemical structure with estrogen. The particular structures contribute to various biofunctions in gynecological disease including cervical cancer and breast cancer. Several flavonoids are reported to exhibit the antitumor effect on cervical cancer cells via suppressing the cell proliferation, metastasis, and invasion [15, 21, 22]. In addition, the TGF*β* signal pathway is well known to promote the carcinogenesis via inducing the EMT pathway and angiogenesis at the advanced stages of tumor [23, 24]. Many compounds could target TGF*β* signal pathway to suppress the cervical cancer. In this study, the inhibitor of the TGF*β* signal pathway was screened from the natural flavonoids using western blot. We found that baicalein exerted the best inhibitory effect on TGF*β* expression. The proliferation and migration tests revealed that baicalein could suppress the proliferation and migration of cells in cervical carcinoma HeLa cells. Additionally, the application of SB431542 revealed that the combination of baicalein and SB431542 reversed the effect of only baicalein on cell proliferation and migration in HeLa cells. These results indicated that the TGF*β* signal pathway might involve in the effect of baicalein against cervical cancer.

Baicalein is a simple-structural flavonoid isolated from traditional Chinese herb of *Scutellaria baicalensis* which was applied to clear away heat and detoxicate in Chinese folk medicine for thousands of years [25, 26]. Inheriting the function of *Scutellaria baicalensis*, baicalein possesses various pharmacological activities such as eliminating inflammation, killing virus, and suppressing oxidant, historically. Many current investigations have also found that baicalein could suppress the different kinds of tumors via the proliferation and apoptosis. Yan et al. revealed that baicalein promoted autophagy and apoptosis of breast cancer cells through the PI3K/AKT pathway [27]. The study by Yu et al. showed that baicalein raised the cisplatin sensitivity to lung adenocarcinoma cells via the PI3K/Akt/NF-*κ*B pathway [28]. It had been reported that baicalein could induce the apoptosis of colon cancer cells through inducing DEPP/Gadd45a and activating MAPKs [29]. The results in this study revealed the inhibitory effect of baicalein on proliferation and migration of cervical cancer HeLa cells via TGF*β* signal pathway. Our findings make an expanding understanding for antitumor effects of baicalein and provide a valuable choice for the treatment to cervical cancer.

The unlimited proliferation is considered as one of the most typical characteristics of malignant tumor. It causes the extreme overgrowth of tumor tissue and predatory behavior to the nutrition and living space of normal tissue, eventually threatening to individual's life. The mTOR/p70S6K signal pathway plays an important role in the regulation of cell proliferation, which involves in the pathological process of cancer, diabetes, cardiovascular disease, and so on. In the tumorigenesis, mTOR protein is activated by the phosphorylation and then phosphorylated its substrate of p70S6K and 4EBP1, leading to the disordered regulation of cell cycle and uncontrolled cell proliferation. Several agents have been identified as an mTOR inhibitor to suppress the tumor [30, 31]. In this study, RT-PCR and western bolt assay displayed that baicalein inhibited the expression and phosphorylation of mTOR, p70S6K, and 4EBP1. Combined with the inhibitory effect of baicalein on cervical cancer HeLa cells proliferation, it is inferred that the mTOR/p70S6K signal pathway might involve in the inhibitory effect of baicalein on proliferation of cervical cancer cells.

Tumor metastasis is another important typical characteristic of malignant tumor, which is considered as the leading death cause of the advance-staged cancer patients. EMT pathway is an essential step of tumor metastasis from primary location to target location [32]. E-cadherin, Snail, and FAK are the three indicators of protein reflecting EMT pathway progression. Among these indicators, E-cadherin, an important member of calcium-dependent transmembrane glycoproteins, maintains the epithelial phenotype. Its decreased expression would trigger the falling of cell's adhesive ability and improvement of migration ability, promoting the EMT progression. Snail, a DNA-binding protein with zinc finger domains, could recognize and bind to the E-box in the promotor of E-cadherin and then inhibit the expression E-cadherin. FAK is a joint molecule gathering the signal protein to the focal adhesion, and its activation would also suppress the expression of E-cadherin. In the study, western blot data showed that baicalein initiated the induction of E-cadherin expression and the inhibition of Snail and p-FAK. These results indicated that baicalein could suppress the migration of cervical cancer HeLa cells via EMT pathway.

## 5. Conclusions

In summary, the present study found that baicalein is an inhibitor of TGF*β* pathway, and it could suppress mTOR/p70S6K pathway-mediated cell proliferation and EMT pathway-related cell migration via TGF*β* pathway in cervical cancer HeLa cells. In this context, we propose a potential mechanism of baicalein suppressing cervical cancer, which lays a theoretical basis for the development of baicalein and the treatment of cervical cancer.

## Data Availability

The data during the current study are available from the corresponding author on reasonable request.

## Supplementary Materials

Supplementary Materials Figure S1: baicalein inhibits the phosphorylation of SAMD2 (a) and SMAD3 (b) in HeLa cells. ^*∗∗*^*p* < 0.01 and ^*∗∗∗*^*p* < 0.001 vs. control group. Figure S2: baicalein suppresses the cell proliferation in SKG IIIa cells via CCK-8 assay. ^*∗*^*p* < 0.05 and ^*∗∗*^*p* < 0.01 vs. control group. Figure S3: baicalein inhibits the phosphorylation level of mTOR (a) and p70S6K (b) in SKG IIIa cells. ^*∗∗*^*p* < 0.01 and ^*∗∗∗*^*p* < 0.001 vs. control group. .
