# Supplementary material for: Antitumor Effects of Baicalein and Its Mechanism via TGFβ Pathway in Cervical Cancer HeLa Cells
Source: Evid Based Complement Alternat Med. 2021 Mar 11;2021:5527190. doi: 10.1155/2021/5527190 (PMC7979304; doi:10.1155/2021/5527190)
Supplement: Supplementary Materials — Figure S1: baicalein inhibits the phosphorylation of SAMD2 (a) and SMAD3 (b) in HeLa cells. ∗∗p < 0.01 and ∗∗∗p < 0.001 vs. control group. Figure S2: baicalein suppresses the cell proliferation in SKG IIIa cells via CCK-8 assay. ∗p < 0.05 and ∗∗p < 0.01 vs. control group. Figure S3: baicalein inhibits the phosphorylation level of mTOR (a) and p70S6K (b) in SKG IIIa cells. ∗∗p < 0.01 and ∗∗∗p < 0.001 vs. control group. . [file 5527190.f1.docx]

**Supplemental Information**

**Anti-tumor effects of baicalein and its mechanism via TGFβ pathway in cervical cancer HeLa cells**

Gang Yu^1, #^, Lizhen Chen^2, #^, Yuanhua Hu^3^, Zhen Yuan^4^, Yao Luo^5^, Yuanhuan Xiong^1, 6, *^

**Figure S1**


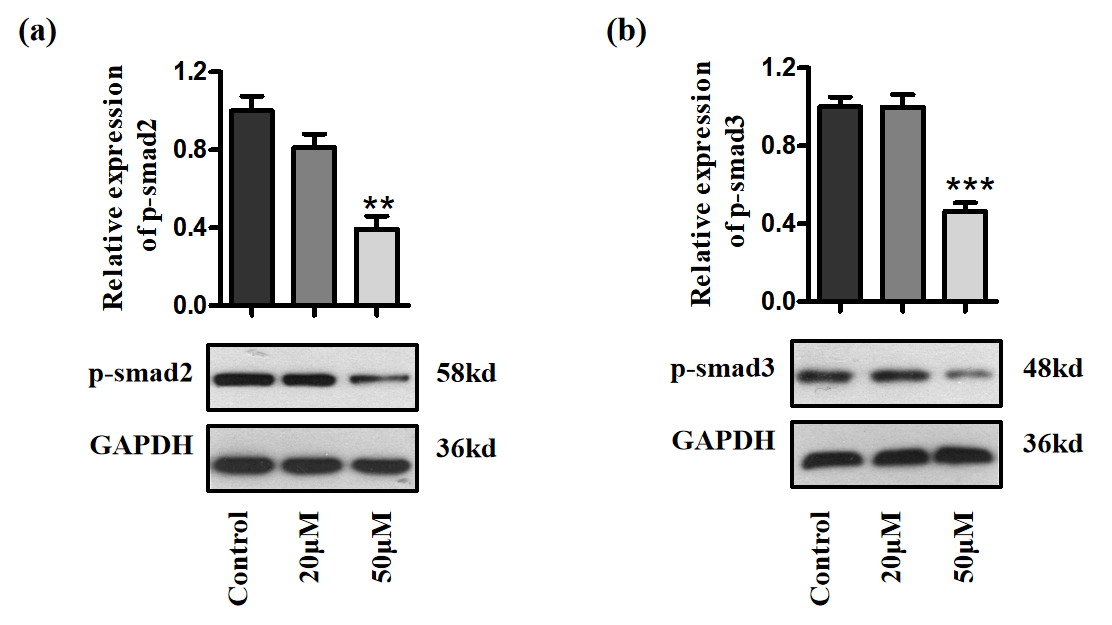


Figure S1 Baicalein inhibits the phosphorylation of smad2 (a) and smad3 (b) in HeLa cells. **p<0.01 and ***p<0.001 v.s. control group.

**Figure S2**

Figure S2 Baicalein suppresses the cell proliferation in SKG IIIa cells via CCK-8 assay. *p<0.05 and **p<0.01 v.s. control group.

**Figure S3**


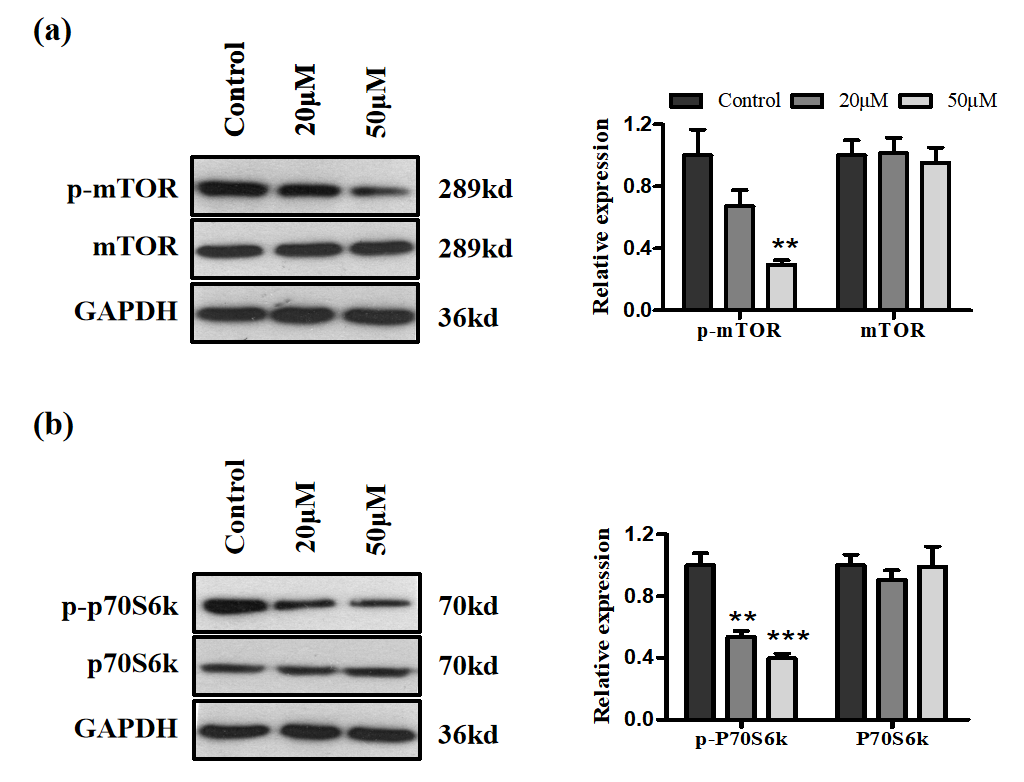


Figure S3 Baicalein inhibits the phosphorylation level of mTOR (a) and p70S6K (b) in SKG IIIa cells. **p<0.01 and ***p<0.001 v.s. control group.
